# Supplementary material for: Nanoscale Dispersion of Carbon Nanotubes in a Metal Matrix to Boost Thermal and Electrical Conductivity via Facile Ball Milling Techniques
Source: Nanomaterials (Basel). 2023 Oct 23;13(20):2815. doi: 10.3390/nano13202815 (PMC10609409; doi:10.3390/nano13202815)
Supplement: Supplementary file 1 [file nanomaterials-13-02815-s001.zip › nanomaterials-2640870-supplementary.docx]

Nanoscale Dispersion of Carbon Nanotubes in a Metal Matrix to Boost Thermal and Electrical Conductivity via Facile Ball Milling Techniques

Bin Li ^1^, Lihua Zhou ^1^, Bo Wang ^1^, Maoshu Yin ^1^, Yong Qian ^1^, Xianglei Shi ^1^, Zhejun Guo ^1^, Zhao Han ^2^, Nantao Hu ^2,^* and Lijie Sun ^1,^*

^1^ Research Center for Photovoltaics, Shanghai Institute of Space Power-Sources, Shanghai 200245, China; tolb10@163.com (B.L.); zhou_lihua1983@163.com (L.Z.); 15522053272@163.com (B.W.);
ymaoshu@163.com (M.Y.); bbqianyong@163.com (Y.Q.); shixianglei99@163.com (X.S.); gzj98762@sjtu.edu.cn (Z.G.)

^2^ Key Laboratory of Thin Film and Microfabrication Technology (Ministry of Education), School of Electronics, Information and Electrical Engineering, Shanghai Jiao Tong University, Dong Chuan Road No. 800, Shanghai 200240, China; zwboss@sjtu.edu.cn

* Correspondence: hunantao@sjtu.edu.cn (N.H.); sunlijielu@163.com (L.S.)


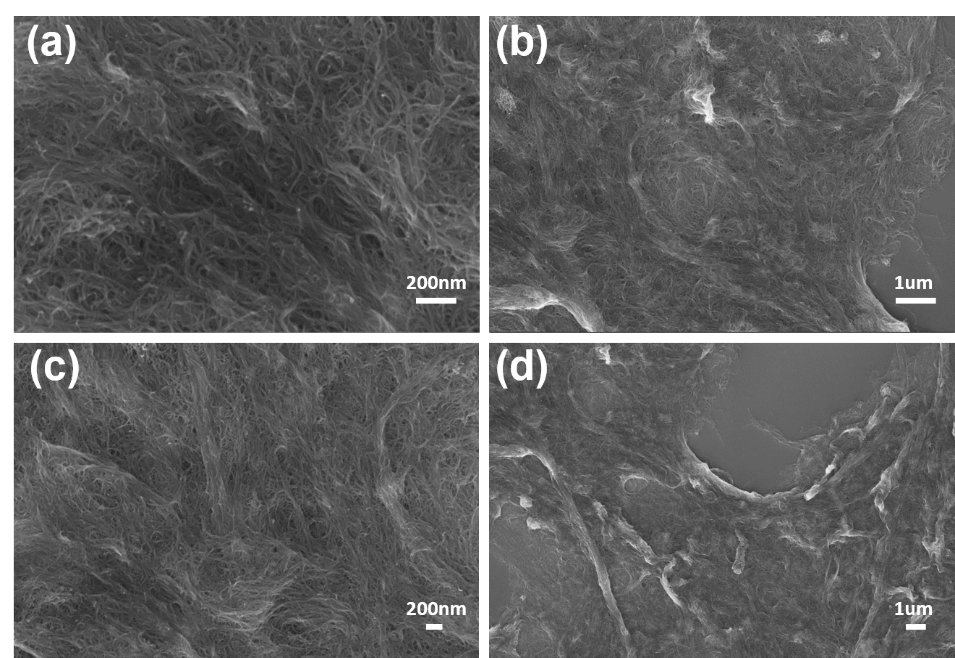


**Figure S1.** (a-d) SEM of CNT at different magnification.


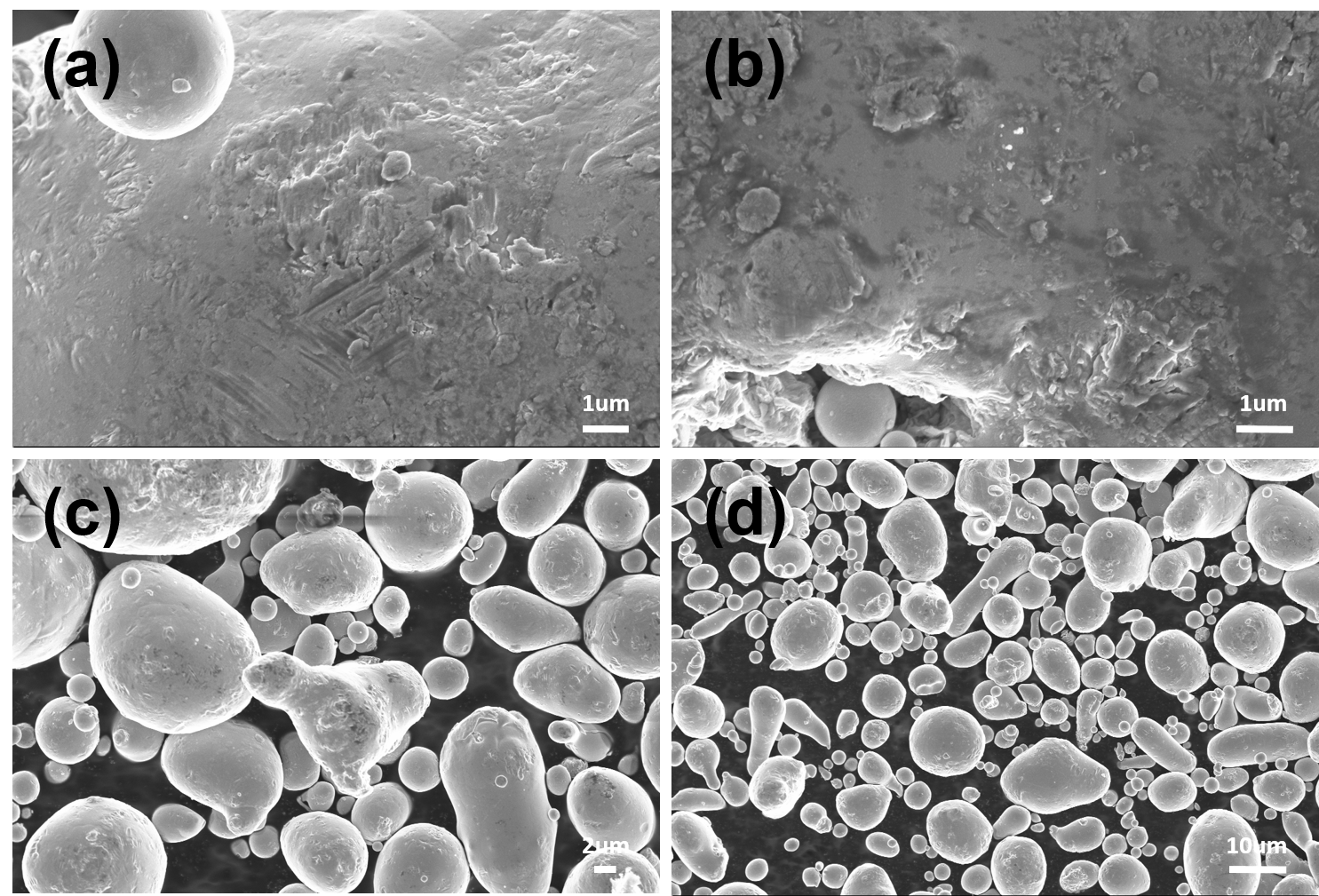


**Figure S2.** (a-d) SEM of Sn at different magnification.


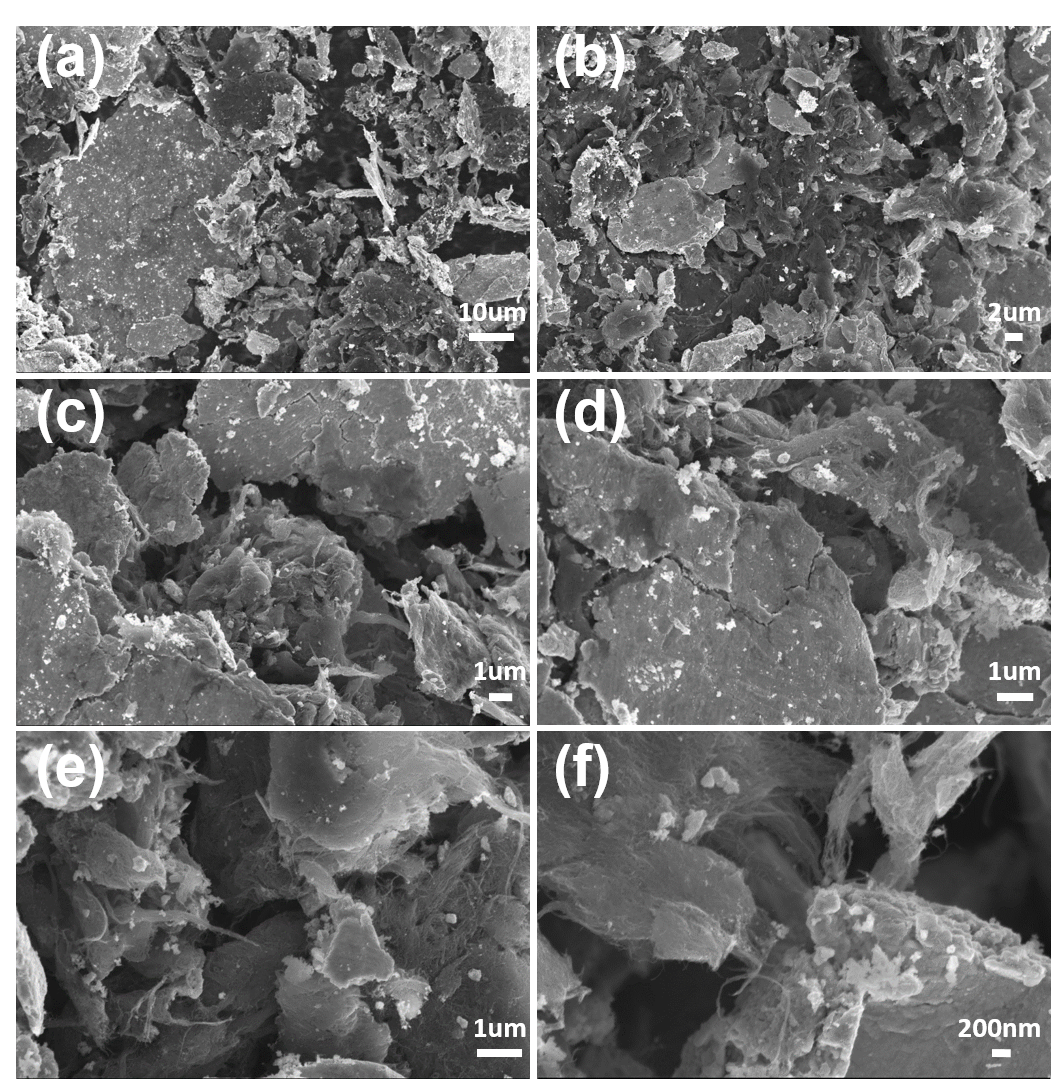


**Figure S3.** (a-f) SEM of CNT-Sn composites with mass fraction of 2 wt.% before sintering at different magnifications.


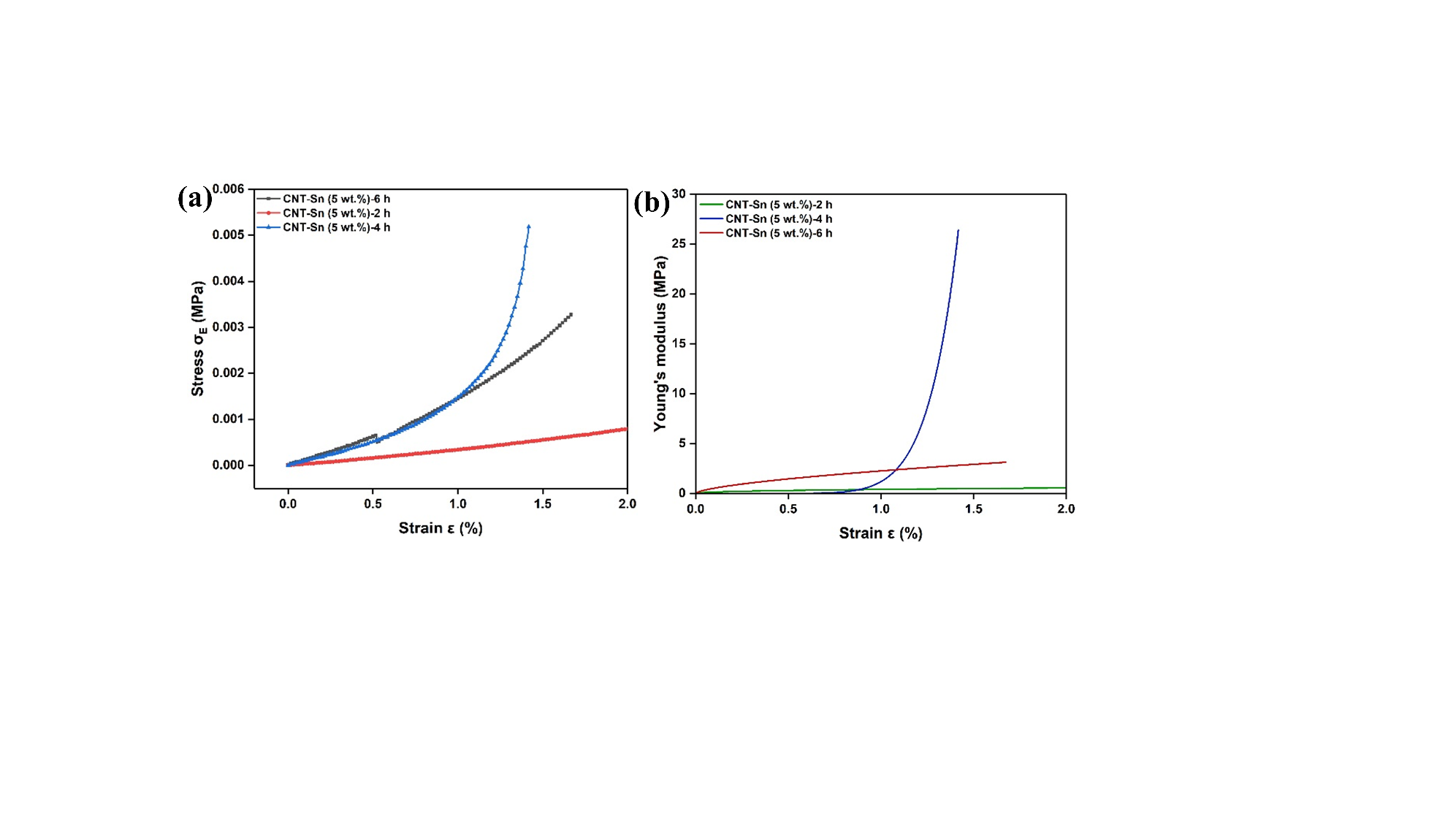


**Figure S4.** (a) Stress strain curves of CNT-Sn composites with 5 wt.% CNT under different ball milling time; (b) Young's modulus curves of sintered CNT-Sn composites with 5 wt.% CNT under different ball milling time.
